# Dengue/dengue hemorrhagic fever: the emergence of a global health problem.

**DOI:** 10.3201/eid0102.952004

**Published:** 1995

**Authors:** D J Gubler, G G Clark

**Affiliations:** National Center for Infectious Diseases, Centers for Disease Control and Prevention, Fort Collins, Colorado, USA.


### Dengue/Dengue Hemorrhagic Fever: The Emergence of a Global Health Problem

Dengue and dengue hemorrhagic fever (DHF) are caused by one of four closely related, but antigenically distinct, virus serotypes (DEN-1, DEN-2, DEN-3, and DEN-4), of the genus *Flavivirus* (1). Infection with one of these serotypes does not provide cross-protective immunity, so persons living in a dengue-endemic area can have four dengue infections during their lifetimes. Dengue is primarily an urban disease of the tropics, and the viruses that cause it are maintained in a cycle that involves humans and *Aedes aegypti*, a domestic, day-biting mosquito that prefers to feed on humans. Infection with a dengue virus serotype can produce a spectrum of clinical illness, ranging from a nonspecific viral syndrome to severe and fatal hemorrhagic disease. Important risk factors for DHF include the strain and serotype of the virus involved, as well as the age, immune status, and genetic predisposition of the patient.

The first reported epidemics of dengue fever occurred in 1779-1780 in Asia, Africa, and North America; the near simultaneous occurrence of outbreaks on three continents indicates that these viruses and their mosquito vector have had a worldwide distribution in the tropics for more than 200 years. During most of this time, dengue fever was considered a benign, nonfatal disease of visitors to the tropics. Generally, there were long intervals (10-40 years) between major epidemics, mainly because the viruses and their mosquito vector could only be transported between population centers by sailing vessels.

A global pandemic of dengue begun in Southeast Asia after World War II and has intensified during the last 15 years. Epidemics caused by multiple serotypes (hyperendemicity) are more frequent, the geographic distribution of dengue viruses has expanded, and DHF has emerged in the Pacific region and the Americas (1,2). In Southeast Asia, epidemic DHF first appeared in the 1950s, but by 1975 it had become a leading cause of hospitalization and death among children in many countries. In the 1980s, DHF began a second expansion into Asia when Sri Lanka, India, and the Maldive Islands had their first major DHF epidemics; Pakistan first reported an epidemic of dengue fever in 1994. The recent epidemics in Sri Lanka and India were associated with multiple dengue virus serotypes, but DEN-3 was predominant and was genetically distinct from DEN-3 viruses previously isolated from infected persons in those countries (3).

After an absence of 35 years, epidemic dengue fever occurred in both Taiwan and the People's Republic of China in the 1980s. The People's Republic of China had a series of epidemics caused by all four serotypes, and its first major epidemic of DHF, caused by DEN-2, was reported on Hainan Island in 1985 (4). Singapore also had a resurgence of dengue/DHF from 1990 to 1994 after a successful control program had prevented significant transmission for over 20 years (5). In other countries of Asia where DHF is endemic, the epidemics have become progressively larger in the last 15 years.

In the Pacific, dengue viruses were reintroduced in the early 1970s after an absence of more than 25 years. Epidemic activity caused by all four serotypes has intensified in recent years with major epidemics of DHF on several islands (6).

Despite poor surveillance for dengue in Africa, we know that epidemic dengue fever caused by all four serotypes has increased dramatically since 1980. Most activity has occurred in East Africa, and major epidemics were reported for the first time in the Seychelles (1977), Kenya (1982, DEN-2), Mozambique (1985, DEN-3), Djibouti (1991-92, DEN-2), Somalia (1982, 1993, DEN-2), and Saudi Arabia (1994, DEN-2) (1,6, CDC, unpublished data). Epidemic DHF has been reported in neither Africa nor the Middle East, but sporadic cases clinically compatible with DHF have been reported from Mozambique, Djibouti, and Saudi Arabia (CDC, unpublished data).

The emergence of dengue/DHF as a major public health problem has been most dramatic in the American region. In an effort to prevent urban yellow fever, which is also transmitted by *Ae. aegypti*, the Pan American Health Organization organized a campaign that eradicated *Ae. aegypti* from most Central and South American countries in the 1950s and 1960s. As a result, epidemic dengue occurred only sporadically in some Caribbean islands during this period. The *Ae. aegypti* eradication program, which was officially discontinued in the United States in 1970, gradually eroded elsewhere, and this species began to reinfest countries from which it had been eradicated. In 1995, the geographic distribution of *Ae. aegypti* was similar to its distribution before the eradication program (Figure 1).

In 1970, only DEN-2 virus was present in the Americas, although DEN-3 may have had a focal distribution in Colombia and Puerto Rico (7). In 1977, DEN-1 was introduced and caused major epidemics throughout the region over a 16-year period (7). DEN-4 was introduced in 1981 and caused similar widespread epidemics (7). Also in 1981, a new strain of DEN-2 from Southeast Asia caused the first major DHF epidemic in the Americas (Cuba) (7).

This strain has spread rapidly throughout the region and has caused outbreaks of DHF in Venezuela, Colombia, Brazil, French Guiana, Suriname, and Puerto Rico. By 1995, 14 countries in the American region had reported confirmed DHF cases (Figure 2), and DHF is endemic in many of these countries.

DEN-3 virus recently reappeared in the Americas after an absence of 16 years. This serotype was first detected in association with a 1994 dengue/DHF epidemic in Nicaragua (8). Almost simultaneously, DEN-3 was confirmed in Panama and, in early 1995, in Costa Rica (8, CDC, unpublished data). In Nicaragua, considerable numbers of DHF were associated with the epidemic, which was apparently caused by DEN-3. In Panama and Costa Rica, the cases were classic dengue fever.

Viral envelope gene sequence data from the DEN-3 strains isolated from Panama and Nicaragua have shown that this new American DEN-3 virus strain was likely a recent introduction from Asia since it is genetically distinct from the DEN-3 strain found previously in the Americas, but is identical to the DEN-3 virus serotype that caused major DHF epidemics in Sri Lanka and India in the 1980s (R. Lanciotti; unpublished data). The new DEN-3 strain, and the susceptibility of the population in the American tropics to it, suggests that DEN-3 will spread rapidly throughout the region and likely will cause major epidemics of dengue/DHF in the near future.

In 1995, dengue is the most important mosquito-borne viral disease affecting humans; its global distribution is comparable to that of malaria, and an estimated 2.5 billion people are living in areas at risk for epidemic transmission (Figure 3). Each year, tens of millions of cases of dengue fever occur and, depending on the year, up to hundreds of thousands of cases of DHF. The case-fatality rate of DHF in most countries is about 5%: most fatal cases are among children.

There is a small, but significant, risk for dengue outbreaks in the continental United States. Two competent mosquito vectors, *Ae. aegypti* and *Aedes albopictus*, are present and, under certain circumstances, each could transmit dengue viruses. This type of transmission has been detected twice in the last 15 years in south Texas (1980 and 1986) and has been associated with dengue epidemics in northern Mexico (7). Moreover, numerous viruses are introduced annually by travelers returning from tropical areas where dengue viruses are endemic. From 1977 to 1994, a total of 2,248 suspected cases of imported dengue were reported in the United States (9, CDC, unpublished data). Although some specimens collected were not adequate for laboratory diagnosis, preliminary data indicate that 481 (21%) cases were confirmed as dengue (9, CDC, unpublished data). Many more cases probably go unreported each year because surveillance in the United States is passive and relies on physicians to recognize the disease, inquire about the patient's travel history, obtain proper diagnostic samples, and report the case. These data underscore the fact that southern Texas and the southeastern United States, where *Ae. aegypti* is found, are at risk for dengue transmission and sporadic outbreaks.

The reasons for this dramatic global emergence of dengue/DHF as a major public health problem are complex and not well understood (10). However, several important factors can be identified. First, effective mosquito control is virtually nonexistent in most dengue-endemic countries. Considerable emphasis for the past 20 years has been placed on ultra-low-volume insecticide space sprays for adult mosquito control, a relatively ineffective approach for controlling *Ae. aegypti*. Second, major global demographic changes have occurred, the most important of which have been uncontrolled urbanization and concurrent population growth. These demographic changes have resulted in substandard

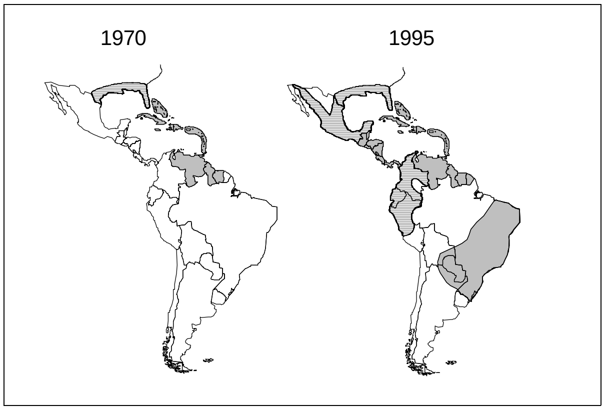

Figure 1. Distribution of *Aedes aegypti* (shaded areas) in the Americas in 1970, at the end of the mosquito eradication program, and in 1995.

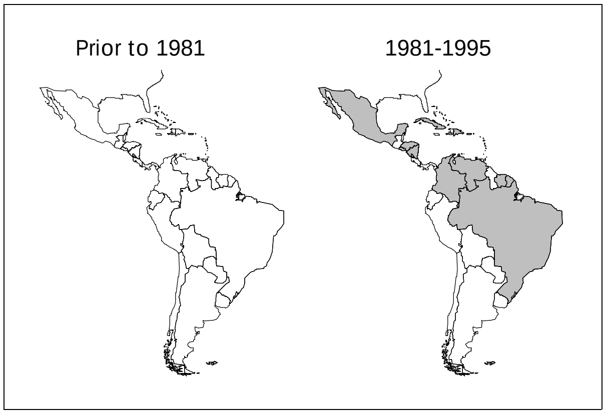

Figure 2. American countries with laboratory-confirmed hemorrhagic fever (shaded areas), prior to 1981 and from 1981 to 1995.

housing and inadequate water, sewer, and waste management systems, all of which increase *Ae. aegypti* population densities and facilitate transmission of *Ae. aegypti*-borne disease. Third, increased travel by airplane provides the ideal mechanism for transporting dengue viruses between population centers of the tropics, resulting in a constant exchange of dengue viruses and other pathogens. Lastly, in most countries the public health infrastructure has deteriorated. Limited financial and human resources and competing priorities have resulted in a "crisis mentality" with emphasis on implementing so-called emergency control methods in response to epidemics rather than on developing programs to prevent epidemic transmission. This approach has been particularly detrimental to dengue control because, in most countries, surveillance is very inadequate; the system to detect increased transmission normally relies on reports by local physicians who often do not consider dengue in their diagnoses. As a result, an epidemic has often reached or passed the peak of transmission before it is detected.

No dengue vaccine is available. Recently, however, attenuated candidate vaccine viruses have been developed in Thailand. These vaccines are safe and immunogenic when given in various formulations, including a quadrivalent vaccine for all four dengue virus serotypes. Unfortunately, efficacy trials in human volunteers have yet to be initiated. Research is also being conducted to develop second-generation recombinant vaccine viruses; the Thailand attenuated viruses are used as a template. However, an effective dengue vaccine for public use will not be available for 5 to 10 years.

Prospects for reversing the recent trend of increased epidemic activity and geographic expansion of dengue are not promising. New dengue virus strains and serotypes will likely continue to be introduced into many areas where the population densities of *Ae. aegypti* are at high levels. With no new mosquito control technology available, in recent years public health authorities have emphasized disease prevention and mosquito control through community efforts to reduce larval breeding sources (11). Although this approach will probably be effective in the long run, it is unlikely to impact disease transmission in the near future. We must, therefore, develop improved, proactive, laboratory-based surveillance systems that can provide early warning of an impending dengue epidemic. At the very least, surveillance results can alert the public to take action and physicians to diagnose and properly treat dengue/DHF cases.

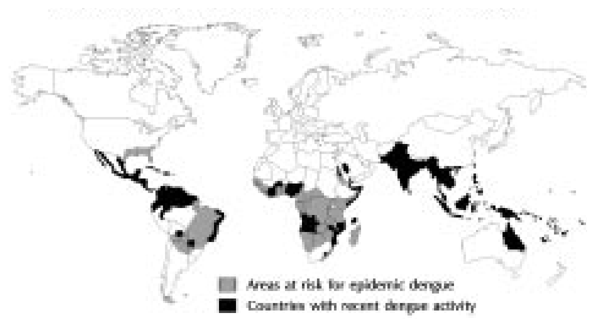

Figure 3. World distribution of dengue viruses and their mosquito vector, *Aedes aegypti*, in 1995.

**Duane J. Gubler and Gary G. Clark**
National Center for Infectious Diseases
Centers for Disease Control and Prevention
Fort Collins, Colorado, and San Juan, Puerto Rico, USA

### References

1. Gubler DJ. Dengue. In: Epidemiology of arthropod-borne viral disease, Monath TPM, editor. Boca Raton (FL): CRC Press, 1988:223-60.
2. Halstead SB. The XXth century dengue pandemic: need for surveillance and research. Rapp Trimest Statist Sanit Mondo 1992;45:292-8.
3. Lanciotti RS, Lewis JG, Gubler DJ, Trent DW. Molecular evolution and epidemiology of dengue-3 viruses. J Gen Virol 1994;75:65-75.
4. Qiu FX, Gubler DJ, Liu JC, Chen, QQ. Dengue in China: a clinical review. Bull WHO 1993;71:349-59.
5. Anonymous. The dengue situation in Singapore. Epidemiol Bull 1994;20:31-3.
6. Gubler DJ. Dengue haemorrhagic fever: a global update [Editorial]. Virus Information Exchange News. 1991;8:2-3.
7. Gubler DJ. Dengue and dengue haemorrhagic fever in the Americas. In: WHO, Regional Office for SE Asia, New Delhi, Monograph. SEARO 1993;22:9-22.
8. Centers for Disease Control and Prevention. Dengue type 3 infection—Nicaragua and Panama, October-November 1994. MMWR 1995;44:21-4.
9. Rigau-Pérez JG, Gubler DJ, Vorndam AV, Clark GG. Dengue surveillance—United States, 1986-1992. MMWR 1994;43:7-19.
10. Gubler DJ, Trent DW. Emergence of epidemic dengue/dengue hemorrhagic fever as a public health problem in the Americas. Infect Agents Dis 1994;2:383-93.
11. Gubler DJ, Clark GG. Community-based integrated control of *Aedes aegypti*: a brief overview of current programs. Am J Trop Med Hyg 1994:50:50-60.
